# The Influence of Maceration on the Biodiversity of Yeasts in the Early Winemaking Stages of White Wine from the Slovak Tokay Wine Region

**DOI:** 10.3390/foods13233792

**Published:** 2024-11-26

**Authors:** Ivana Regecová, Jana Výrostková, Boris Semjon, Viera Lovayová, Pavlina Jevinová, Zuzana Megyesy Eftimová, Martin Bartkovský, Monika Pipová, Slavomír Marcinčák

**Affiliations:** 1 Department of Food Hygiene Technology and Safety, University of Veterinary Medicine and Pharmacy in Košice, Komenského 73, 041 81 Košice, Slovakia; ivana.regecova@uvlf.sk (I.R.); jana.vyrostkova@uvlf.sk (J.V.); pavlina.jevinova@uvlf.sk (P.J.); zuzana.megyesy.eftimova@uvlf.sk (Z.M.E.); martin.bartkovsky@uvlf.sk (M.B.); monika.pipova@uvlf.sk (M.P.); slavomir.marcincak@uvlf.sk (S.M.); 2 Department of Medical and Clinical Microbiology, Faculty of Medicine, Pavol Jozef Šafárik University in Košice, Trieda SNP 1, 040 11 Košice, Slovakia; viera.lovayova@upjs.sk

**Keywords:** diversity, fermentation, maceration, Tokay, wine, yeast

## Abstract

This study investigates the effect of maceration and different winemaking techniques on the species diversity of yeasts in white wines from the Slovak Tokay wine region, known for its traditional white wine production. Lipovina grape variety samples were divided into three groups: control (C), macerated (M) and macerated with the addition of a yeast culture (MY). During the entire fermentation process, quantitative and qualitative microbiological analyses of the raw material and must samples were carried out, which resulted in the identification of 60 yeast isolates via the API 20 C AUX biochemical test and MALDI-TOF MS. Identification was further verified via Sanger sequencing of PCR amplicons, which confirmed the presence of less common wild yeasts in Tokay wine must samples, including *Aureobasidium pullulans*, *Cryptococcus magnus*, *Torulaspora delbrueckii* and *Rhodotorula* sp. The highest species diversity was observed in the macerated group. These findings indicate that the quality and distinctiveness of Slovak Tokay wines can be increased by careful management of the maceration process during winemaking procedures.

## 1. Introduction

Tokay wines are recognized as the most famous Slovak commodity, with a protected designation of origin. Their importance goes beyond commercial value and they deserve the status of a national treasure with cultural value. They are produced in one of the oldest and smallest wine-growing regions in the world—the Slovak Tokay wine region [1]. The Tokay wine region in Slovakia includes seven villages situated within close proximity to each other (up to 13 km): Bara, Čerhov, Černochov, Malá Tŕňa, Slovenské Nové Mesto, Veľká Tŕňa and Viničky, with volcanic rocks and tuff subsoil. The distinctive microclimate in the Tokay region (fog along with warm and dry days) combined with specific grape varieties (e.g., Lipovina) and enological techniques sets this wine region apart from all others. The diversity of mycobiota in the terroir originating primarily from the vineyard and influenced by various factors such as the climate, topography and season is also crucial for high quality wine production [2,3,4,5]. Furthermore, the winemaking process, which consists of grape ripening, harvesting, processing, alcoholic fermentation and wine aging, can significantly affect the microbiota of the wine during the production cycle [6]. There is a great interest in characterizing the microbial community, especially yeast species other than *Saccharomyces* spp. Traditional microbiological techniques have provided important information about the mycological diversity in the pro-environment of vineyards [7,8]. *Rhodotorula* spp., *Cryptococcus* spp., *Candida* spp. and *Aureobasidium pullulans* have been shown to predominate on unripe grape berries. On the other hand, *Hanseniaspora*/*Kloeckera* spp. and *Metschnikowia* spp. are observed to predominate on the surface of ripe grapes [9]. When the grapes are disrupted, the yeast population increases, with a predominance of *Hanseniaspora/Kloeckera* spp., *Metschnikowia* spp., *Candida* spp., *Saccharomyces* spp. and *Zygosachcaromyces* spp. [6,10]. Certain genera of yeasts, especially *Candida*, *Pichia* and *Hanseniaspora*, colonize the area where the stem is attached to the berry. These yeast genera are found to be present during grape ripening up until harvest. In particular, they predominate at the beginning of spontaneous fermentation and provide an alternative mechanism by which the environment can affect the wine quality and flavor [11,12,13]. The geographical area, together with the climate and year, prove to be an important determinant for most mycobiota, especially at the beginning of the fermentation process [14].

Due to the growing demand for quality Tokay wines and changing consumer preferences, it is necessary to diversify the white wine market [15]. Wines that are characterized by unique organoleptic properties are constantly more in demand, which encourages producers to incorporate new and modified traditional enological procedures into their production [16]. Maceration is one such traditional procedure. It refers to the period during which the grape solids (skins, seeds and stems) are in contact with the grape juice [17]. When different maceration techniques are used, the cell walls of the grape skins are disrupted, which promotes the transfer of various compounds into the must, including aromatic substances, and can increase antioxidant activity, thereby affecting the aromatic properties of the wine [18,19,20]. Prolonged skin contact is commonly applied to improve the extraction of components, including mycobiota, into the must, thus contributing to the overall improvement of wine quality [21]. According to Peinada et al. [22] and Radeka et al. [23], pre-fermentation maceration is used to enhance the varietal character of white wines, resulting in the creation of balanced, rounder and stronger wines. This procedure allows for a slight increase in the phenolic fraction, as the absence of ethanol keeps the concentration of phenols at a low level, preventing excessive astringency and bitterness [23].

The extraction of phenolic compounds during maceration and alcoholic fermentation occurs due to the degradation of the pectin layer of the cell wall and depends on the degree of ripeness of the grapes [24]. Late harvesting is typically used to produce dessert wines with a higher content of reducing sugars [25].

This process also affects the pH of the wine, reducing its acidity and increasing the concentration of volatile acids. The sensory profile of white wines subjected to maceration is characterized by higher aromatic intensity due to the extraction of aromatic volatile compounds and their precursors, along with a typical orange or amber color [26,27,28,29]. At the same time, these wines are enriched with compounds such as ethyl acetate, TDN (1,1,6-trimethyl-1,2-dihydronaphthalene) and ethyl-3-methylbutyrate, which contribute to their floral and fruity tones and their sweet perception [26,27]. A higher concentration of benzoic acid can add notes of honey, dried figs and tobacco [27].

Maceration allows for the maximization of the potential of both the grapes and their mycobiota, resulting in wines that are not only refreshing but also interesting to consumers. There are only a few studies that investigate the impact of maceration on the diversity of mycobiota and the quality of Tokay wines [30,31,32]. Therefore, this study focuses on identifying the yeast species present during fermentation in different winemaking procedures (longer maceration, longer maceration with the addition of yeast culture, fermentation without maceration with yeast culture) for the production of Lipovina variety Tokay wines of Slovak origin.

## 2. Materials and Methods

### 2.1. Winemaking and Sampling Procedures

The monovarietal white grape variety Lipovina (*Vitis vinifera* L.) from the Slovak Tokay wine region was manually harvested and transported to an experimental wine cellar in the University of Veterinary Medicine and Pharmacy in Košice (Košice, Slovakia). The grapes were harvested in a good sanitary state and at optimal maturity, with soluble solids (sugar content) ranging from 21.5 to 23.5° Brix. Subsequently, the grapes were destemmed and crushed using standard equipment in the experimental wine cellar. The whole volume of grapes was destemmed and crushed at once. Subsequently, the entire volume was divided into three parts for each treatment separately. The first experimental group (control (C)) was distributed into stainless steel tanks with a volume of 100 L, to which sulfur dioxide (SO_2_) was added at a concentration of 40 mg/L and the yeast culture *Saccharomyces cerevisiae* without pre-fermentative skin maceration (O.K. SERVIS BioPro, s.r.o., Prague, Czech Republic) in the amount of 30 g/100 L (approximately 3 × 10^3^ cfu/mL). The second experimental group (M) was subjected to a maceration process in a stainless steel tank for 7 days without access to oxygen at a temperature of 18 ± 2 °C with SO_2_ added in the amount of 40 mg/L. After maceration, the fermented must was pressed and transferred to a stainless steel tank where the fermentation process was completed. The third group (MY) underwent the same process as the second experimental group; however, the yeast culture Saccharomyces cerevisiae (O.K. SERVIS BioPro, s.r.o, Praha, Czech Republic) was added in the amount of 30 g/100 L (approximately 3 × 10^3^ cfu/mL) during the 7 day maceration process. Each experimental treatment was performed in triplicate. The fermentation processes of each group took place at a controlled temperature (18 ± 2 °C) for four weeks. Investigated samples were taken from raw and non-treated grapes (RMs) and, subsequently, during the vinification processes. Samples (100 mL) were taken on days 0, 3, 7 and 14 and in week 4, put into sterile closable containers and immediately microbiologically examined.

### 2.2. Microbiological Examination Procedures

The preparation of the basic suspension and subsequent decimal dilutions for the microbiological culture solution was performed according to ISO standard 6887-1 [33]. The prepared dilutions were combined with 18 ± 2 mL of PCA agar broth (Hi-Media, Maharashtra, India) and cooled to 44–47 °C, then used for total viable count (TVC) determination. The inoculated and solidified agar plates were incubated at 30 ± 1 °C/72 h. After incubation, colonies were counted both on the surface and within the Petri dishes. Plates containing between 10 and 300 colonies were selected for microorganism quantification, and the results were adjusted to reflect the concentration of microorganisms in 1 mL of the original sample, in accordance with ISO standard 4833-1 [34].

In order to create a comprehensive basic microbiological profile, the amount of lactic acid bacteria (LAB) was also determined in addition to the TVC. LAB were isolated using three successive dilutions, each of which was spread with 0.1 mL of the sample onto the surface of De Man, Rogosa and Sharpe agar (Hi-Media, Maharashtra, India), a selective diagnostic medium. All inoculated plates were incubated under anaerobic conditions at 37 °C/48 h using an AnaeroGen bag (Oxoid, Hampshire, UK). Only plates with colony counts between 10 and 150 were considered for LAB quantification.

Yeast quantitative analysis followed ISO Standard 21527-1 [35]. The first three decimal dilutions were each inoculated in 0.1 mL onto the surface of Dichloran Rose Bengal Chloramphenicol (DRBC) agar (Hi-Media, Maharashtra, India), which had a final pH of 5.6 ± 0.2. The plates were then incubated at 25 °C for 5 days. Plates with fewer than 150 colonies were selected for colony counting, and 5 colonies with similar phenotypic characteristics were isolated for further analysis. All microbiological culture procedures were conducted simultaneously. All microbiological culture examinations were carried out in parallel.

Prior to species identification, the selected colonies were re-inoculated on the surface of DRBC agar to ensure the purity of the colony, which was necessary for accurate identification. Contaminated strains were excluded from species identification.

### 2.3. Yeast Species Identification

#### 2.3.1. Identification Based on Biochemical Reactions

The identification of individual yeast species was first performed using API 20 C AUX tests (Biomerieux, Marcy-l’Étoile, France). Individual yeast strains were inoculated from the DRBC agar with a sterile bacterial loop onto the surface of the Sabouraud agar (Hi-Media, Maharashtra, India) and incubated at a temperature of 25 °C for 5 days. After incubation of the inoculated plates, the strains were resuspended on the day of the analysis in the API 20C AUX medium, which is part of the diagnostic kit. Subsequently, the test strains prepared in this way were applied to the test plates according to the manufacturer’s instructions. The inoculated test plates were incubated at a temperature of 30 °C for 48 h–72 h. The results were processed and evaluated using Apiweb^TM^ API 20C AUX V5.0 software (Biomerieux, Marcy-l’Étoile, France). As part of the identification accuracy evaluation, the Apiweb^TM^ API 20C AUX V5.0 software used statistical indicators, namely the sum of %id and the sum of the T indexes. The sum of %id is the sum of the relative proximity of the biochemical profile of the tested strain to the different taxa of the database (%id). In this way, it is possible to determine whether the biochemical profile obtained is close to the taxon or not. The sum of the T indexes is the sum of the proximity to the most typical profile in each of the taxa (T index). The most typical taxon is the one that has no tests against the identification in relation to the percentages shown in the database for the taxon in question.

#### 2.3.2. Matrix-Assisted Laser Desorption Ionization Time-of-Flight Mass Spectrometry

The identification of yeast species was first conducted using matrix-assisted laser desorption ionization time-of-flight mass spectrometry (MALDI-TOF MS). This method involves comparing the protein mass fingerprint of the unknown organism with those in a reference database by following the standard sample preparation protocol by Bruker Daltonics [36], which utilizes formic acid and acetonitrile. The analysis was carried out on an Ultra-flex III instrument (Bruker, Billerica, MA, USA) with the assistance of Flex Analysis software (version 3.0). For the evaluation, BioTyper software (version 1.1) from Bruker was employed. After the evaluation process, species identification was completed using various methods.

#### 2.3.3. Polymerase Chain Reaction and Sequencing

Subsequently, the species identification of yeasts was confirmed by polymerase chain reaction (PCR) according to White et al. [37]. The genomic DNA of the tested yeast isolates was extracted for the given analysis using a modified method with the commercially available E.Z.N.A.^®^ Fungal DNA mini kit (OMEGA Biotek, Norcross, GA, USA). A 10–50 mg sample of the biomass was taken from the Czapek agar medium using a sterile scalpel and transferred to a 1.5 mL micro test-tube. The sample was first exposed to a low temperature (−20 °C) for 5 min and then heated to 95 °C for 5 min. This freezing and heating cycle was repeated three times. Subsequently, the DNA was isolated using the commercial isolation kit, and 10 µL of proteinase K (Macherey-Nagel, Düren, Germany) was added to the micro test-tubes containing the mycelium and the mixture was incubated at 37 °C for 30 min. DNA isolation was then performed according to the manufacturer’s instructions in the commercial kit. A mixture of silica and glass beads (a ratio of 1:1) along with 10 µL of proteinase K were added to a mycelial sample (10–50 mg) in a micro test-tube and the samples were then incubated at 37 °C for 30 min. Next, after vigorous vortexing of the samples for 1 min, 800 µL of lysis solution FG1 from the kit was added to the micro test-tube and the samples were incubated at 65 °C for 10 min while simultaneously being exposed to ultrasonic waves (500 Hz). DNA isolation was completed according to the recommended procedure of the isolation kit. DNA purity and concentration were evaluated with a spectrometer BioSpec-nano (SHIMADZU, Wien, Austria). The resulting supernatant was used as a source of DNA for the PCR reactions.

The ITS1 and ITS4 primers for amplification of the ITS region were synthesized and used following the protocol described by White et al. [37]. Amplification was performed in a total volume of 20 μL, using HOT Firepol^®^ Blend Master Mix (Amplia, s.r.o., Bratislava, Slovakia), containing 1.0–10 ng DNA, with each primer at a concentration of 10 pmol/μL. The reaction was performed in a thermocycler (CFX96 system, Biorad, Hercules, CA, USA) with the following cycling conditions: an initial denaturation at 95 °C/13 min, followed by 20 s at 95 °C for DNA denaturation, 60 s at 53 °C for annealing and 2 min at 72 °C for extension. The amplification process was completed by cooling the reaction to 6 °C. The resulting PCR products were sequenced using the Sanger method by SEQme s.r.o. (Dobříš, Czech Republic). Sequence data were uploaded to the GenBank-EMBL database for comparison with sequences in the National Center for Biotechnology Information (NCBI) nucleotide database, accessible via http://www.ncbi.nlm.nih.gov/BLAST (accessed on 4 June 2024).

## 3. Results

### 3.1. Microbiological Examination of Cultured Samples

**Figure 1:**
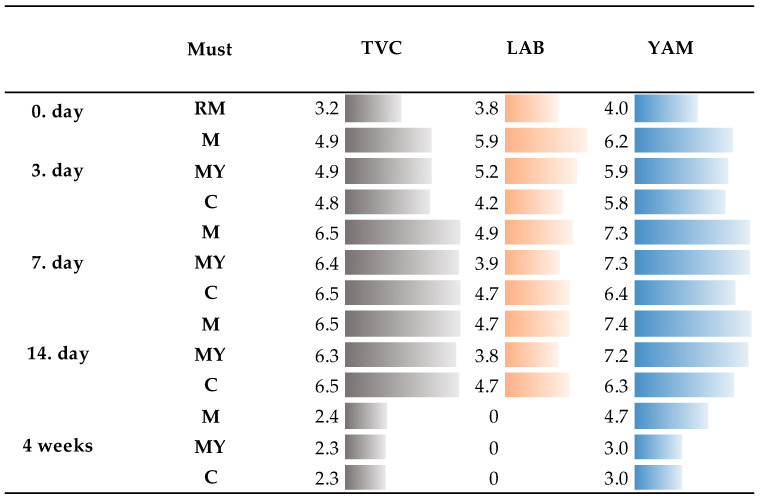
A graphic representation of the results of the microbiological examination of the cultured samples of the raw material (log cfu/g) and musts during the fermentation process (log cfu/mL). RM—raw material; M—macerated must; MY—must with the addition of a yeast culture; C—control must; TVC—total viable count (sd: ±0.1); LAB—count of lactic acid bacteria (sd: ±0.0); YAM—count of yeasts and molds (sd: ±0.1).

The quantification of yeasts and molds (YAM), lactic acid bacteria (LAB) and the total number of microorganisms (TVCs) was carried out by microbiologically examining the cultured samples. As shown in Figure 1, the TVC was determined in the raw material samples to be 3.2 ± 0.1 log cfu/g, which was lower than the count of lactic acid bacteria and yeasts in the raw material. This situation can be explained by the specific cultivation conditions for each microbial group, such as different incubation temperatures, incubation times and modified environments for LAB cultivation (microaerophilic conditions).

Subsequently, the TVC values in other must samples increased until the seventh day of fermentation, when the given numbers in all the samples reached their maximum (6.4–6.5 ± 0.1 log cfu/mL), which remained stabilized until the fourth week of fermentation, when an important decrease in the number of TVCs in all the groups was observed.

When determining the number of LAB in the input raw material samples, the numbers belonged to the level 3.8 ± 0.0 log cfu/g. After the third day of the fermentation process, the values increased; however, in the fourth week of fermentation, LAB were not detected to be present in any of the experimental groups.

During the microbiological examination of the samples, the amount of YAM in the raw material was also determined, which proved to be 4.0 log ± 0.1 cfu/g. After the 3rd day of fermentation, there was a sharp increase in the number of YAM in all samples, reaching a maximum on the 7th day of fermentation, which was maintained until the 14th day of fermentation (6.3–7.4 ± 0.1 log cfu/mL). In the fourth week of fermentation, there was a significant decrease in the number of YAM, especially in the experimental group MY and C.

When comparing the results of the quantitative microbiological examination be-tween the individual experimental groups of musts, small differences were observed. Higher numbers of LAB were detected in experimental group must M after the 3rd, 7th and 14th days of the fermentation process. The highest number of YAM was determined on the seventh day of fermentation for all groups. However, in group M, higher numbers of YAM were detected during the whole observation period.

### 3.2. Yeast Species Identification

Subsequently, after the microbiological culture examination of the must samples, the species identification of 60 isolated yeasts (with different phenotypic expressions) was performed based on the biochemical profile, MALDI-TOF MS and the PCR method, followed by sequencing of the obtained PCR amplicons from the ITS region.

When identifying yeasts using the API 20 C AUX test as an initial screening method for the investigated mycobiota, the obtained biochemical profiles were compared with taxon profiles in the Apiweb^TM^ API 20C AUX V5.0 software database. According to the test results, the isolates were assigned to the genera *Candida*, *Cryptococcus*, *Kloeckera*, *Pichia*, *Rhodotorula* and *Saccharomyces*.

As shown in Table 1, the sum of the %id and the sum of the T indexes indicated the accuracy of species identification based on biochemical profiles either as excellent identification (%id ≥ 99.9 and T ≥ 0.75), very good identification (%id ≥ 99.0 and T ≥ 0.50), good identification (%id ≥ 90.0 and T ≥ 0.25) or acceptable identification (%id ≥ 80.0 and T ≥ 0). Based on these values, isolates of the species *Saccharomyces cerevisiae* and *Rhodotorula glutinis* were the most accurately identified. Good identification was demonstrated for the genus *Kloeckera* and species such as *Candida magnoliae*, *Candida utilis* and *Pichia membranifaciens*. In some cases, the %id values ranged from ≥80.0 and the T index was 0, indicating a less reliable identification at the species level.

Since discrimination at the species level was not sufficient, identification using MALDI-TOF MS was conducted as an additional rapid identification method. Table 1 demonstrates that not all isolates were identified. Some of the isolates that were identified using the API test as strains of the genera *Candida*, *Kloeckera* and *Rhodotorula nothofagi* were not identified using MALDI-TOF MS and had a score value of less than 1.60, indicating an unreliable identification even at the genus level.

When comparing the results of identification by both methods, they appeared to be very reliable identification methods for isolates identified as *Saccharomyces cerevisiae* and *Rhodotorula glutinis,* where the score value and the sum of the %id confirmed a reliable identification. On the other hand, in the case of *Candida utilis* isolates, the identification appeared to be reliable when using the API test based on the obtained values but the score value from MALDI-TOF MS ranged from 1.69 to 1.82, indicating an unreliable identification or a reliable identification only at the genus level. *Hanseniospora uvarum* isolates, on the other hand, were not identified using the API test but the score value ranged from 2.03 to 2.53, confirming a reliable identification at the species level. A similar situation occurred in the identification of the species *Cryptococcus magnus,* where the API test did not identify the species but the MALDI-TOF MS did, although with a lower score value.

As mentioned above, species discrimination was low for some isolates; therefore, further identification at the molecular level using the PCR method and subsequent sequencing of the obtained amplicons was carried out, by which the genera *Candida*, *Cryptococcus*, *Metschnikowia*, *Pichia*, *Rhodotorula*, *Saccharomyces*, *Torulaspora* and *Aureobasidium* were identified (Table 1). In total, out of 18 identified species and 1 genus, 17 species were identified by PCR and sequencing, 5 species of which were not identified by MALDI-TOF MS and 7 species were either not identified or were not correctly identified by the API test. Strains identified by the API 20C AUX test as *Kloeckera* spp. (the sum of the %id. ≥ 90.0; the sum of the T indexes ≥ 0.25) were confirmed as *Hanseniaspora uvarum* by sequencing. Strains identified by the API 20C AUX test as *Candida famata* (the sum of the %id. ≥ 99.0; the sum of the T indexes ≥ 0.50) and *Candida magnoliae* (the sum of the %id. ≥ 90.0; the sum of the T indexes ≥ 0.25) were confirmed by sequencing as *Pichia kudriavzevii* and *Pichia kluyveri*. When comparing the accuracy of identification using MALDI-TOF MS and the PCR method, it was confirmed that, even with a lower score, the identification was reliable at the genus level. Our results show that the identification by MALDI-TOF MS with a lower score coincided with the identification by the PCR method. Strains identified by the API 20C AUX test as *Kloeckera* spp., *Candida famata* and *Candida magnolia* were not identified by MALDI-TOF MS. After comparing all three identification methods, a retrospective percentage evaluation of individual must samples was carried out, where the results of the PCR identification and subsequent sequencing of the obtained amplicons were taken into account.

### 3.3. Yeast Species Variability During Fermentation

**Figure 2:**
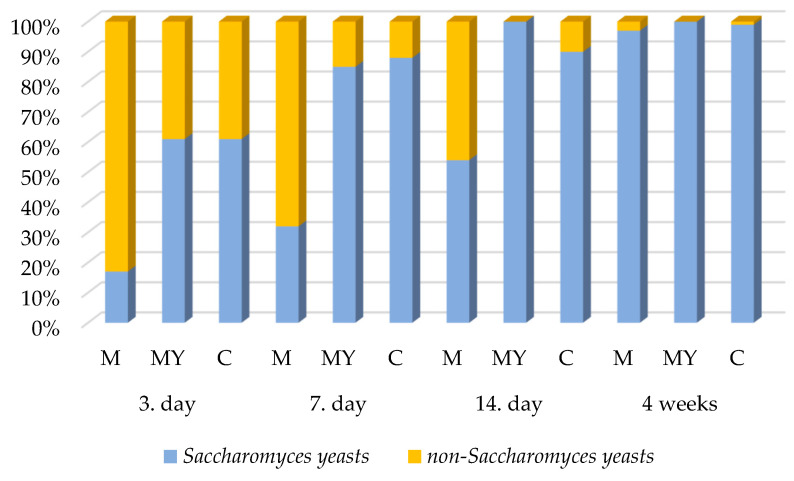
A graphic representation of the quantity of Saccharomyces yeasts and non-Saccharomyces yeasts present in individual must samples expressed as a percentage. RM—raw material; M—macerated must; MY—must with the addition of a yeast culture; C—control must.

The graphic presentation of the results (Figure 2) shows us that, at the beginning of the fermentation process of all the types of must (the third and seventh day), the presence of non-*Saccharomyces* yeasts prevailed. However, with the continuation of the fermentation process, the situation changed and, in the last observed phase, yeasts of the genus *Saccharomyces* prevailed. Non-*Saccharomyces* yeasts (3%) were detected in the final stages of fermentation, especially in the M must samples.

At the same time, non-*Saccharomyces* yeasts, especially *Hanseniospora uvarum* and *Metschnikowia pulcherrima* species, were detected to a greater extent in the raw material samples. The presence of less frequent yeasts such as *Aureobasidium pullulans*, *Cryptococcus magnus*, *Pichia kluyveri*, *Pichia membranifaciens*, *Rhodotorula glutinis* and *Torulaspora delbrueckii* was also detected. In a more specific overview of the quantitative representation of the species in the must samples, a greater diversity of non-*Saccharomyces* yeasts was observed in the M samples up to the fourth week of fermentation, where the species *Hanseniospora uvarum*, *Metschnikowia pulcherrima* and *Pichia kluyveri* were still detected. The yeast *Candida zeylanoides* was detected in the smallest amount and only up to the third day of the fermentation process and that only in the M and MY samples. A low percentage was also detected for the species *Torulaspora delbrueckii* in the macerated must up to the seventh day of fermentation. In the MY and C musts, the species diversity was significantly reduced after the 14th day of fermentation (Table 2).

*Saccharomyces cerevisiae* dominated the analyzed samples until the fourth week of the fermentation process. *Hanseniospora uvarum* and *Metschnikowia pulcherrima* were present in a high percentage in the early stages (the third and seventh day); however, they gradually decreased in number. Yeasts of the genus *Candida* (e.g., *Candida tenuis* and *Candida parapsilosis*) and *Cryptococcus magnus* were present in a very low percentage. The species *Pichia kluyveri* was present up to week 4 but its quantity decreased in favor of the dominant yeast.

In sample M, which underwent maceration, a greater species diversity of yeasts was detected from the third day of the fermentation process and they persisted longer than in the other samples. In addition to *Saccharomyces cerevisiae*, *Hanseniospora uvarum* (in groups M and C), *Metschnikowia pulcherrima* (M) and *Pichia kluyveri* (M) were also detected up to the fourth week of fermentation although in very low percentages.

The final comparison of the species biodiversity of all the experimental groups of musts showed that the greatest biodiversity was present in the macerated must. In the must with an added yeast culture, the biodiversity was reduced already after the third day of fermentation. In the must without the addition of yeast and without maceration, the biodiversity of yeasts gradually declined until the 14th day of fermentation.

## 4. Discussion

The fermentation processes and the associated biodiversity of yeasts play a key role in the production of Tokay wines known for their unique taste and aroma. Therefore, in our study, for the initial detection and monitoring of the development of the microbial community during the first stages of the fermentation process, conventional microbiological examinations of cultured samples of different groups of musts from Lipovina grapes of the Slovak Tokay wine region were used, namely a group with controlled fermentation, a group that underwent maceration and a third group that underwent maceration and had a *Saccharomyces cerevisiae* yeast culture added to it.

The microbiological examination of the cultivated samples was aimed at determining the TVC and the amount of LAB and YAM. In the raw material, the TVC was determined to be 3.2 ± 0.1 log cfu/g. The amount of TVCs rose to a maximum on the seventh day of fermentation, which remained stabilized until the fourth week of fermentation. The amount of LAB in the raw material reached 3.8 ± 0.0 log cfu/g; however, after 4 weeks of fermentation its presence was not detected in any of the samples. The amount of YAM in the raw material was 4.0 log ± 0.1 cfu/g, and the maximum values were also determined to be on the seventh day of fermentation. Higher amounts of YAM were detected in the macerated must M, even in the fourth week of fermentation, compared with the groups with the addition of a yeast culture. The reason may be the slower alcoholic fermentation in the must that underwent maceration, as non-*Saccharomyces* yeasts were still present, which are less efficient in the production of ethanol. A similar study dealing with the mycobiota of Tokay wines was carried out by [9], where they observed a TVC of 2.48 log cfu/g to 4.52 log cfu/g in the input raw material used for the production of Tokay wines. Similarly, in the study of Malomo et al. [38], the TVC in the wine fermentation process increased from 3.311–6.834 log cfu/mL at the beginning to 5.571–9.076 log cfu/mL at the end of storage. The alcoholic fermentation of grape must for wine is a dynamic process, which also induces changes in the microbial population throughout fermentation. It is a complex microbiological process in which various microorganisms participate, including, in addition to the mentioned yeasts, LAB. The presence of these microorganisms during fermentation leaves characteristic traces that depend on their duration and degree of dominance during this process [39]. Yeasts play an essential role in alcoholic fermentation and are usually divided into two main groups: *Saccharomyces* and non-*Saccharomyces*. Non-*Saccharomyces* yeasts begin to multiply at the beginning of fermentation, as they have a lower tolerance to ethanol. However, *Saccharomyces cerevisiae*, which is able to tolerate higher concentrations of ethanol and grows faster, gradually recedes [6]. Non-*Saccharomyces* species originate predominantly from the vineyard environment, such as soil, vine and grape surfaces, and their presence is influenced by various factors such as geographical location, climatic conditions, grape variety and viticultural practices [14].

In our study, biochemical tests and molecular methods were used to determine the representation of individual yeast species expressed as a percentage during the fermentation process of all the tested types of must from the Lipovina order. Work dealing with the identification of the biodiversity of yeasts has evolved slowly in the recent decades in terms of the techniques utilized. Such work requires knowledge and specific tools. The prevailing methods were based on classical morpho-physiological tests according to Barnett et al. [40]. Most authors reported studies on the macroscopic features of colonies [41]. They used rapid biochemical tests such as the API test [42]. In our study, the API 20 C AUX test was utilized for the initial identification step. Several genera and species were confirmed, including *Kloeckera* spp., *Candida famata* and *Candida mognoliae,* which, however, were not confirmed by further investigations. Inconsistencies with correct identification have also been confirmed by several studies, where they commonly detected differences between the results of these tests [43]. In addition, Kačániová et al. [9] confirmed that the API 20CAUX test did not yield satisfactory results in the identification of some types of wine yeast species; *Hanseniaspora uvarum* species were incorrectly identified, for example. At the same time, the isolates were identified using the API 20C AUX test as *Candida krusei*, *Rhodotorula minuta*, *Candida magnoliae* and *Candida famata/Saccharomyces cerevisiae*, while sequencing of the ITS region identified them as *Pichia spp.*, *Saccharomycopsis vini*, *Torulaspora delbrueckii* and *Metschnikowia spp.* Similar misidentifications have been reported in other studies [44,45]. This discrepancy may arise from the fact that *Torulaspora* spp. and *Metschnikowia* spp. are not included in the Apiweb™ database, thus preventing their identification with this system. Consequently, alternative methods, such as MALDI-TOF MS, have been employed in other studies for the accurate identification of these species [46]. This method, along with other molecular analyses, came into use later on for the identification of wine yeasts such as in the study by Gaspar et al. [47] in the Sebeș vineyard (Apollo-Blaj center), followed by Găgeanu et al. [41] in the Dealurile Bujorului vineyard and Dumitrache et al. [48] in the center of Pietroasa (Dealu Mare vineyard); these results were also linked to the sequencing data by Rădoi-Encea et al. [49]. Therefore, in our study, we also utilized MALDI-TOF MS as a more accurate screening method for the identification of the wine yeast species present. Since the results of the API test and MALDI-TOF MS differed in the identification of some yeast species, identification using PCR amplicon sequencing of the ITS region was conducted. The following species were confirmed in Lipovina grape berries via this method: *Aureobasidium pullulans, Candida parapsilosis, Candida tenuis, Candida utilis, Candida zeylanoides, Cryptococcus magnus, Hanseniospora uvarum, Metschnikowia pulcherrima, Pichia kluyveri, Pichia kudriavzevii, Pichia membranifaciens, Rhodotorula glutinis, Rhodotorula nothofagi, Saccharomyces bayanus, Saccharomyces cerevisiae* and *Torulaspora delbrueckii*. Kačániová et al. [9] also identified yeast species such as *Metschnikowia pulcherrima, Rhodotorula glutinis* and *Saccharomyces cerevisiae* in this grape variety. *Metchnikowia pulcherrima* belonged amongst the most dominant yeast species of the Tokay grapes examined, which was confirmed by the study of Kántor et al. [50], which found that its representation in the raw material was 5%. These findings were also confirmed by Fugelsang and Edwards [51], who confirmed the presence of the species *Hansenula anomala, Kluyveromyces marxianus, Candida stellata, Debaryomyces* spp. and *Dekkera* spp., which were not present in our samples, justified by a different terroir. The influence of terroir on the biodiversity of yeasts on grape berries has also been confirmed by our previous study from 2022, which mainly dealt with the mycobiota in the Sobran region—an Eastern Slovak wine-growing region, where similar species were confirmed such as *Candida tenuis, Pichia kudriavzevii, Pichia kluyveri, Pichia fermentas, Metschnikowia pulcherrima, Hanseniospora uvarum, Torulaspora delbrueckii* and *Saccharomyces cerevisiae* but in different percentages [52]. In the Tokay wine region in Hungary, the most widespread species of yeasts are *Metschnikowia pulcherrima* and *Candida stellata* (recently differentiated into two species—*C. stellata* and *C. zemplinina*). These two species of *Candida* especially appear to be typical yeasts of Tokay aszú berries, after the aszú grapes have been harvested, transported and stored. A study by Torija et al., 2002, conducted in the Priorat region, detected a slightly different biodiversity of yeasts, with the main species being *S. cerevisiae*, *Aerobasidium pullulans* and *Hanseniaspora uvarum*, along with *C. zemplinina* [31,53].

*Hanseniaspora uvarum* and *Metschnikowia pulcherrima* are the main species that predominate on grapes in the Tokay wine-growing region. It is well documented that the yeast *Kloeckera apiculata* dominates Slovak vineyards (it is an anamorph of *Hanseniaspora uvarum*) [54,55]. The predominant species in unripe grapes are *Rhodotorula* spp., *Cryptococcus* spp. and *Candida* spp. [50]. On the contrary, *Metchnikowia pulcherrima* and *Hanseniospora uvarum* were the predominant species present in grapes used in our study.

*Metschnikowia pulcherrima* has a variety of beneficial properties related to its enological properties such as low alcohol tolerance (so that a lower alcohol yield can be achieved in the early stages of the fermentation process), a good ability to produce esters, support of glycerol production and inhibition of the presence of *Brettanomyces* spp. and LAB [56].

Non-*Saccharomyces* yeasts were also identified in the initial stages of fermentation, especially in the macerated group. In the groups with controlled fermentation (C) and macerated must with the addition of a yeast culture (MY), these yeasts were present to a lesser extent. Currently, the importance of natural yeasts and the need for their subsequent isolation and selection is increasingly emphasized. Recent research confirms that non-Saccharomyces can have a significant contribution and are even key to achieving wines with exceptional organoleptic and sensory properties. In Europe, a number of studies have been carried out that support the use of unconventional yeasts in the fermentation of grape must. Considering the diverse properties and biotechnological roles of these yeasts, it is essential to develop new approaches for their effective use during grape fermentation. This has also been confirmed by a study that identified 500 different yeasts belonging to 12 species of the non-*Saccharomyces* group out of a total of 568 yeasts isolated from spontaneously fermented musts. The most dominant species was *Haseniospora uvarum,* which accounted for 55% of all isolates, followed by *Metschnikowia pulcherrima,* representing 16.6% of the population. Other non-*Saccharomyces* yeast species identified at a lower frequency (<4%) included *Torulaspora delbrueckii* and *Rhodotorula mucilaginosa* [57,58].

*Torulaspora delbrueckii* is a yeast with a high fermentation potential, which ranks among the important non-*Saccharomyces* species and is used in mixed fermentations with *Saccharomyces cerevisiae*. Research shows that its presence in fermentation can intensify the aroma of wine and improve its overall quality characteristics [59]. One of the main mechanisms of its competitiveness against *Saccharomyces cerevisiae* is a high ethanol tolerance, along with the ability to produce lethal toxins, the concentration of which depends on the particular strain [60,61]. Other useful yeast species, such as the genus *Hanseniaspora*, are known to contribute to the increase in volatile acidity in wine and can promote the formation of acetate esters when used in mixed fermentation with *Saccharomyces cerevisiae* [62,63]. *Hanseniaspora* spp. are characterized by the production of active enzymes such as β-d-glucosidase, which distinguishes them from other yeast species and allows them to contribute to the aromatic properties of wine and improve alcoholic fermentation in the early stages [64]. Several studies have reported a significant increase in the concentration of important aromatic compounds and an improvement in wine color in the mixed fermentation of *Hanseniaspora uvarum* and *Saccharomyces cerevisiae* [65,66]. Although *Hanseniaspora* spp. dominate the initial stages of fermentation, details of their metabolism and ability to adapt to biotic pressure are still lacking. Research focused on nutrient management and analysis of the transcriptome and metabolome of *Hanseniaspora* spp. in the presence of other non-*Saccharomyces* will help to better understand the complex interactions between these species. Another yeast identified in our study was *Cryptococcus magnus,* despite the fact that the genus *Cryptococcus* was reported to be one of the few typical non-*Saccharomyces* yeast species present on the surface of grapes, confirmed also by several studies [67,68,69,70,71]. These yeasts are not present throughout the whole fermentation process; however, they are able to use a wide range of carbon sources at the beginning of fermentation [72]. It was detected only in a low quantity in our experimental groups of musts, which may have been caused by the incubation temperature being 18 °C, while its optimal growth temperature is 25 °C. Isolates of the species *Rhodotorula nothofagi* were also identified. Their origin was detected by Sampaio [73] in decayed wood or seawater. As a result, the author noted that the ecology of the species still remains unclear; however, several strains of *Rhodotorula nothofagi* were isolated from the white musts of Verdicchio, Italy and Rebula, Slovenia [74,75]. In 2009, *Rhodotorula nothofagi* was also associated with Veltline green grapes in small quantities. Based on the findings known so far, it can be assumed that *Rhodotorula nothofagi* is related to strains isolated from European white grape varietals [76].

Non-*Saccharomyces* yeasts, however, are not able to complete alcoholic fermentation, which is why the sequential inoculation of grapes must have been recently employed. Thus, non-*Saccharomyces* yeasts can be inoculated at the beginning of fermentation and, after the fermented must attains about a 10% ethanol content, *Saccharomyces* yeasts are added. In this way, fermentation is completed by the *Saccharomyces* species and the non-*Saccharomyces* species produce the necessary metabolites to positively affect the aroma of the wine. In summary, sequential fermentations with *Saccharomyces* and non-*Saccharomyces* yeast species allow for the development of local wines with a low alcohol content [77].

Many wine makers resort to traditional winemaking procedures such as maceration. Maceration contributes to differences in the development of microbiota compared with conventional winemaking. The unavailability of oxygen causes a slow development of the characteristic yeasts of enological transformation and helps the development of the natural characteristic mycobiota, whose biodiversity is closely linked to the terroir of the grapes [78].

This has also been confirmed by our study, in which only the samples from the macerated group had higher numbers of YAM throughout the entire fermentation process; the highest values of YAM appeared on the seventh day of fermentation. The M musts showed a higher yeast species diversity from the third day of fermentation and maintained this diversity longer than the other samples. Although the dominant *Saccharomyces cerevisiae* predominated, species such as *Hanseniospora uvarum* and *Metschnikowia pulcherrima* appeared in the macerated samples up to the fourth week of fermentation.

There are only a few studies that show the impact of these practices on the mycobiota present during enological fermentation for the production of white wine. Mycobiota in the process of fermentation associated with maceration are influenced by two variables, namely the availability of oxygen and the concentration of sugar. At the same time, the mycobiota is influenced by the effects of the physical treatment of the grape bunch after harvesting, which can affect the rate of diffusion of the components of the grape berries and, thus, the availability of nutrients such as sugar and nitrogenous substances for the yeasts. The homogenization of the grape mass and the subsequent maceration support the availability of such components for the yeasts and also stimulate their activity and growth [78]. Fermentation, which already takes place during maceration, leads to alcohol production, malic acid degradation, pectolytic and proteolytic reactions and the formation of volatile compounds and the diffusion of phenolic substances from the skin to the must [79].

Although pre-fermentation treatment should theoretically have no effect on ethanol content, some studies have suggested that 10 days of maceration can cause an increase in ethanol content of more than 0.5% by volume [80], and a significant increase in dry matter of 13–22% compared with samples of wine that have not undergone maceration. This increase in dry matter is expected, since various compounds and minerals are extracted from the solid part of the grape berries during 48 h of maceration [81].

During the vinification processes associated with maceration, yeasts produce an increased amount of higher alcohols, which result from the conversion of branched-chain amino acids [82]. These higher alcohols are significant contributors to wine aroma, and their biosynthesis is likely influenced by the type or strain of yeast used [83].

It is the direct and easy availability of fermentable carbon sources during maceration that gives the possibility of easier growth, even to more growth-demanding yeasts, before *Saccharomyces cerevisiae* yeast takes over the main role in the fermentation process. The greater presence of *Saccharomyces cerevisiae*, after the addition of yeast culture, supports the rapid accumulation of ethanol, which subsequently acts as an inhibitor of the growth of other types of yeast, thereby reducing their biodiversity [84]. This phenomenon is also confirmed by the findings of our study, where a faster reduction in biodiversity was recorded in the groups of ciders with added yeast culture. In the test group where only maceration was performed, a greater biodiversity of yeasts in the fermentation process was confirmed; probably, the longer contact with grape skins and the absence of yeast cultures contributed to the preservation of a wider microbial diversity.

It should be added that biodynamic agricultural practices may raise some doubts among scientists, as these practices are not always supported by sufficient scientific evidence. Nevertheless, in the context of winemaking practice, such an approach can maximize the contribution of the native mycobiota to defining wine characteristics [85].

The results of our research confirm the claims of Patrignani et al. [85], and Karlsson and Karlsson [86] that grape processing methods such as prolonged maceration can significantly affect the development of yeast communities during fermentation. At the same time, a biodynamic approach that does not use yeast starters can contribute to a greater biodiversity of mycobiota, which in turn has a significant impact on the sensory properties and quality of the wine.

## 5. Conclusions

Maceration, although traditionally used mainly in the production of red wine, also plays a role in the production process of white wine, as pointed out in our study. The seven-day maceration of the experimental must group affected the species biodiversity of the yeasts; they displayed higher numbers of non-*Saccharomyces* yeasts during fermentation. Their presence in this process and their isolation and selection can enrich the quality of wine production. Identification methods for the detection of yeast biodiversity play an important role in the field of winemaking; therefore, in our study we utilized a traditional method of identification—the API 20 C AUX test—and modern approaches—MALDI-TOF MS and sequencing. It has been found that, while the API test can provide rapid results, it often leads to the inaccurate identification of yeast species that are not sufficiently covered in the database. On the contrary, MALDI-TOF MS and sequencing offer more accurate and reliable identification, which is essential for the understanding of the complexity of the mycobiota at different stages of the fermentation process. This advance in identification technologies represents a fundamental step in the optimization of fermentation processes and the overall quality of wine. Terroir, as a sum of natural factors where geographical, climatic and soil conditions create a unique environment that shapes the character and taste of Tokay wines, also largely influences the biodiversity of yeasts in the Tokay vineyards. Various methods of vinification, including maceration, allow for an increased diversity and complexity in the resulting products, thus contributing to the uniqueness of wines from this region.

## Figures and Tables

**Table 1:** Yeast species identification from samples of the raw material and musts.

	Number ITS Sequence in	API 20C AUX Test		MALDI-TOF MS
	Gen Bank	Sum of the %id	Sum of the T Indexes	(Score Value)
*Aureobasidium pullulans*	KU301224.1	-	-	
	OP758081.1			1.75–1.8
*Candida parapsilosis*	OR528193.1	≥80.0	0	- *
*Candida tenuis*	HQ283371.1	≥80.0	0	1.82–1.9
*Candida utilis*	AF218990.1	≥90.0	≥0.25	1.69–1.82
*Candida zeylanoides*	KR089867.1	≥80.0	0	1.86–2.03
*Cryptococcus magnus*	JQ425371.1	-	-	1.95–2.22
	KX078429			
*Hanseniospora uvarum*	MT635299.1	-	-	
	PP481703.1			2.03–2.53
	OR507597.1			
*Metschnikowia pulcherrima*	OR475099.1	-	-	1.85–2.31
	MW506809.1			
*Pichia kluyveri*	DQ104732.1	≥80.0	0	1.72–1.99
*Pichia kudriavzevii*	KX023221.1	≥80.0	0	1.82–2.00
	OP269746.1			
*Pichia membranifaciens*	KU687363.1	≥90.0	≥0.25	1.78–1.83
	MK310154.1			
*Rhodotorula glutinis*	OR435441	≥99.9	≥0.75	2.10–2.32
*Rhodotorula nothofagi*	AB038096.1	≥80.0	0	- *
*Saccharomyces bayanus*	AZ124408.1	≥80.0	0	1.65–1.92
*Saccharomyces cerevisiae*	OR554112.1			
	JF505283.1	≥99.9	≥0.75	2.00–2.19
	AM262827.1			
*Torulaspora delbrueckii*	OR435441	-	-	1.99–2.29
	OR145787.1			

*—isolates using MALDI-TOF MS were not species-identified and their score value was below 1.60.

**Table 2:** The percentage of individual types of yeast species present during the fermentation process of different types of must.

Fermentation	0. Day	3. Day			7. Day			14. Day			4 Weeks			
Sample	RM	M	MY	C	M	MY	C	M	MY	C	M	MY	C	
*Aureobasidium* *pullulans*	1%	3%	1%	-	5%	-	-	4%	-	-	-	-	-	
*Candida tenuis*	1%	5%	2%	4%	4%	-	-	3%	-	-	-	-	-	
*Candida parapsilosis*	1%	1%	1%	-	2%	-	-	1%	-	-	-	-	-	
*Candida zeylanoides*	1%	2%	1%	-	-	-	-	-	-	-	-	-	-	
*Candida utilis*	2%	1%	-	2%	2%	-	-	-	-	-	-	-	-	
*Cryptococcus magnus*	1%	5%	2%	1%	1%	-	-	-	-	-	-	-	-	
*Hanseniospora uvarum*	3%	13%	9%	10%	15%	7%	3%	16%	-	5%	1%	-	1%	
*Metschnikowia* *pulcherrima*	3%	15%	10%	10%	14%	7%	7%	13%	-	3%	1%	-	-	
*Pichia kluyveri*	2%	11%	7%	5%	12%	-	2%	4%	-	2%	1%	-	-	
*Pichia kudriavzevii*	1%	7%	2%	-	2%	-	-	1%	-	-	-	-		
*Pichia membranifaciens*	2%	5%	2%	2%	4%	-	-	3%	-	-	-	-	-	
*Rhodotorula glutinis*	2%	4%	1%	-	3%	-	-	1%	-	-	-	-	-	
*Rhodotorula nothofagi*	1%	4%	-	2%	2%	-	-	-	-	-	-	-	-	
*Saccharomyces* *cerevisiae*	2%	14%	60%	60%	32%	83%	88%	54%	100%	90%	97%	100%	99%	
*Saccharomyces bayanus*	1%	3%	1%	1%	-	3%	-	-	-	-	-	-	-	-
*Torulaspora delbrueckii*	3%	7%	1%	3%	2%	-	-	-	-	-	-	-	-	-

RW—raw material; M—macerated must; MY—must with the addition of a yeast culture; C—control must.

## Data Availability

The original contributions presented in this study are included in the article, further inquiries can be directed to the corresponding author.
